# Effects of Dexmedetomidine in Improving Oxygenation and Reducing Pulmonary Shunt in High-Risk Pediatric Patients Undergoing One-Lung Ventilation for Thoracic Surgery: A Double-Blind Randomized Controlled Trial

**DOI:** 10.7759/cureus.69659

**Published:** 2024-09-18

**Authors:** Ayham Khddam, Faten Rostom, Mohammad Y. Hajeer

**Affiliations:** 1 Department of Anesthesia and Resuscitation, Children's Hospital, University of Damascus, Damascus, SYR; 2 Department of Anesthesia, Faculty of Medicine, University of Damascus, Damascus, SYR; 3 Department of Orthodontics, Faculty of Dentistry, University of Damascus, Damascus, SYR

**Keywords:** dexmedetomidine, pulmonary shunt fraction qs/qt, one-lung ventilation, pulmonary vasoconstriction, hypoventilation

## Abstract

Background and objectives

Pediatric thoracic surgery has unique considerations due to the immaturity of the respiratory system anatomically and physiologically, which presents technical and pharmacological considerations, including the very common technique of one-lung ventilation (OLV), which causes serious complications in children. Therefore, we investigated the effects of dexmedetomidine on oxygenation and pulmonary shunt fraction (Qs/Qt) in high-risk pediatric patients undergoing OLV for thoracic surgery. This randomized controlled trial aimed to investigate dexmedetomidine's effect on the partial pressure of arterial oxygen (PaO_2_) and pulmonary shunt fraction (Qs/Qt).

Methods

A total of 63 children underwent thoracic surgery with OLV and were divided into two groups. The dexmedetomidine group (group Dex, n = 32) received dexmedetomidine (0.4 μg/kg/hour), and the placebo group (group placebo, n = 31) received normal saline. Two arterial and central venous blood samples were taken for arterial and venous blood gas analysis at four time points: T1 (10 minutes after mechanical ventilation of total lung ventilation), T2 (10 minutes after OLV), T3 (60 minutes after OLV), and T4 (20 minutes after the end of OLV). At these intervals, the following parameters were measured: PaO_2_, Qs/Qt, mean arterial pressure (MAP), heart rate (HR), and peak inspiratory pressure (PIP).

Results

The two groups had no significant differences in FEV1/FVC and baseline pulmonary shunt fraction (Qs/Qt). Dexmedetomidine significantly improved PaO_2_ compared with placebo during OLV (T2 and T3). There was a significant decrease in Qs/Qt compared with placebo during OLV (T2, T3, and T4). There was a decrease in PIP compared with placebo during OLV (T2 and T3). No statistically significant differences in MAP or HR were observed between the groups.

Conclusion

Infusion of dexmedetomidine during OLV in high-risk pediatric thoracic surgery reduces shunt and pulmonary shunt fraction Qs/Qt, improves PaO_2_ and body oxygenation, reduces PIP and pressure load, and maintains hemodynamic stability (MAP, HR).

## Introduction

The pediatric cardiovascular system's unique anatomical and physiological characteristics include smaller ventricular chambers and reduced systolic mass [1]. Furthermore, pediatric physiology is characterized by decreased systemic vascular resistance [2], coupled with a dominant parasympathetic nervous system influence [3]. This slower response in mean arterial pressure (MAP) compared to heart rate (HR) variations can be attributed to the influence of the dominant parasympathetic nervous system, leading to a delayed effect [4].

Each terminal bronchiole opens into a single alveolus in the pediatric respiratory system, forming primordial acinar groups rather than the fully developed alveolar sac clusters seen in adults. These immature acinar groups undergo progressive growth and development, eventually achieving a mature, adult-like structure by approximately six to eight years [5]. Ventilation in children primarily relies on the movement of the diaphragm and respiratory rate. Respiratory mechanics in children is also characterized by a more horizontal diaphragm position, resulting in reduced mechanical efficiency [6]. Children also exhibit increased chest wall compliance, contributing to their characteristically higher respiratory rates [7]. This, coupled with a lower functional residual capacity (FRC) compared to adults, predisposes children to pulmonary atelectasis and the development of shunts [6].

While frequently employed in thoracic surgery for enhanced surgical field visualization, one-lung ventilation (OLV) poses a significant physiological challenge in pediatric patients. The disruption of the ventilation/perfusion (V/Q) ratio induced by OLV can lead to increased intrapulmonary shunting and an elevated pulmonary shunt fraction (Qs/Qt), thereby increasing the risk of hypoxemia in children undergoing thoracic procedures [8]. The pulmonary shunt fraction (Qs/Qt) represents the proportion of deoxygenated blood flow bypassing the alveoli, calculated as the ratio of shunt flow (Qs) to the total pulmonary blood flow (Qt) [9]. In contrast, the FEV1/FVC ratio, a key measure of pulmonary function, represents the proportion of the forced vital capacity (FVC) that can be exhaled within the first second (FEV1) [6].

Hypoxic pulmonary vasoconstriction (HPV) is the primary compensatory mechanism against regional lung hypoxia. By diverting pulmonary blood flow away from poorly ventilated alveoli towards well-ventilated areas, HPV aims to optimize ventilation-perfusion matching, thereby mitigating the effects of hypoxia and improving gas exchange [10]. The efficacy of HPV is, however, influenced by various factors, including alterations in pulmonary artery pressure, alkalosis, the administration of vasodilators, and, most notably, the use of inhaled anesthetic agents [10]. This study was designed as a two-group controlled clinical trial using propofol, which is known not to affect HPV [11].

Dexmedetomidine, a widely used anesthetic adjuvant, exerts its effects through selective activation of alpha-2 adrenergic receptors. Binding to presynaptic alpha-2 receptors inhibits norepinephrine release, while activation of postsynaptic alpha-2 receptors suppresses sympathetic nervous system activity. This ultimately results in reductions in both blood pressure and HR [12]. This alpha-2 adrenergic stimulation can lead to vasodilation and subsequent hypotension. Furthermore, alpha-2 adrenergic blockade can effectively suppress pulmonary arrhythmias responsive to adrenergic stimulation, although it does not influence those arising from hypoxemia and hypoventilation [13]. Given the complex effects of dexmedetomidine on the cardiovascular system, further investigation is warranted to elucidate its influence on intrapulmonary shunt and HPV in patients undergoing OLV [14]. This study hypothesized that dexmedetomidine administration would improve arterial oxygenation, as measured by the partial pressure of arterial oxygen (PaO_2_), and attenuate pulmonary shunting during OLV in pediatric patients.

## Materials and methods

Study design and settings

This study employed a rigorous methodology, using a single-center, randomized controlled trial with a parallel-group design. Participants were randomly allocated in a 1:1 ratio to the intervention and control groups. The double-blind nature of the study ensured that neither the participants nor the researchers were aware of the treatment allocation throughout the study, minimizing potential bias and enhancing the reliability of the findings. The trial was approved by the Local Research Ethics Committee of Damascus University Children's Hospital (Approval No.: 1810b/3.2024). The trial was registered in the ClinicalTrials.gov registry (NCT06505772). This study was conducted at Damascus University Children's Hospital over a period of 13 months, commencing on May 1, 2023, and concluding on June 1, 2024.

Sample size calculation

The sample size was estimated based on a previous trial on the same topic [15], in which Qs/Qt was the primary outcome measure of the study; therefore, Qs/Qt at the T3 time point of OLV-60 minutes in the reference was chosen as the index for sample size calculation. The mean ± SD value of Qs/Qt in the dexmedetomidine group (DEX group) was 21.76 ± 3.34, and the mean ± SD value of Qs/Qt in the saline group was 27.57 ± 1.30. We performed the power analysis using G*Power 3.1.9.7 software. When the power was set at 95% and the alpha level was set at 5%, the required sample size was 30 patients for each group. The total number of patients was increased to 36 patients to compensate for any drop-outs.

Inclusion criteria

This study investigated male and female children aged four to six years undergoing thoracic surgery requiring OLV. Inclusion criteria stipulated an American Society of Anesthesiologists (ASA) physical status classification of III [16], indicating severe systemic disease, specifically thoracic and respiratory conditions. Furthermore, participants were required to have a forced expiratory volume in 1 second to forced vital capacity ratio (FEV1/FVC) between 75% and 80% and a pulmonary shunt fraction (Qs/Qt) exceeding 10%, reflecting the standard criteria employed within research. Children patients meeting these criteria were eligible for inclusion.

Exclusion criteria

Several exclusion criteria were applied to ensure participant safety and homogeneity. Patients with pre-existing comorbidities, including neurological illnesses, congenital or valvular heart diseases, hepatic dysfunction, and renal failure, were excluded. Intraoperatively, individuals who did not achieve satisfactory lung isolation, hindering the effective application of OLV, were also excluded. Additionally, participants experiencing inadequate oxygen saturation (SpO_2_ < 90%) and unresponsive to standard anesthetic interventions aimed at improving oxygenation, necessitating abandonment of the OLV procedure, were excluded. Finally, individuals presenting with bradycardia (HR < 80 beats per minute) refractory to atropine administration or requiring inotropic support (e.g., dopamine) were excluded from the study.

Interventional group (DEX group) protocol

This study employed a two-arm interventional design. Children scheduled for thoracic surgery requiring OLV under general anesthesia were randomly allocated to either the DEX group or the control group. The DEX group received an intravenous infusion of dexmedetomidine at a rate of 0.4 μg/kg/hour, while the control group received a comparable volume of normal saline.

Before anesthesia induction, all children, regardless of group assignment, received a 2 mL/kg bolus of normal saline. Standard monitoring procedures were implemented for all participants, encompassing noninvasive blood pressure (NIBP), pulse oximetry for oxygen saturation monitoring, electrocardiography, temperature assessment, end-tidal carbon dioxide (EtCO_2_) monitoring, invasive arterial blood pressure (IBP) monitoring, and bispectral index (BIS) monitoring to assess the depth of anesthesia. Anesthetic induction included intravenous fentanyl (1 µg/kg) and propofol (1 mg/kg) intravenously until BIS values reached 50 (40-50) according to each child, followed by atracurium 0.5 mg/kg and endotracheal intubation. Dexmedetomidine was injected into the first group, and saline was injected into the second group.

Dexmedetomidine administration protocol

Dexmedetomidine administration in the DEX group followed a specific protocol. Initially, a loading dose of dexmedetomidine was administered intravenously at a rate of 0.5 μg/kg/hour for 10 minutes. Following this loading dose, the infusion rate was adjusted based on the individual child's weight to maintain a continuous infusion of dexmedetomidine at 0.4 μg/kg/hour. This maintenance infusion was continued throughout the surgical procedure until skin closure, at which point the infusion was discontinued.

Control group (placebo group) protocol

The placebo group received the same amount of saline instead of dexmedetomidine, using the same protocol.

One-lung ventilation procedure

In this study of pediatric participants (aged four to six years), bronchial intubation, verified by careful auscultation with a stethoscope before and after lateral positioning, successfully achieved OLV during thoracic surgery. This technique was preferred over double-lumen tubes (DLTs), usually reserved for children older than eight years (minimum size 26 Fr), and provided satisfactory lung isolation, preventing the need for bronchial blockers. While DLTs have been used successfully in some children weighing 18 kg or more, bronchial intubation has proven to be a suitable alternative for our younger study population.

Following the opening of the pleural cavity, OLV was achieved by inserting a double-lumen endotracheal tube. Proper tube placement was meticulously verified via auscultation with a stethoscope before and after positioning the patient in the lateral decubitus position. This ensured accurate isolation of the operative lung and adequate ventilation of the non-operative lung. Contralateral bronchoalveolar intubation is performed for children with small-gauge isolation tubes that are not available. The surgical procedure was performed after opening the chest and using the retractor to achieve complete lung isolation. A multi-lumen central venous catheter was placed through the right or left internal jugular vein, depending on the side of the surgery, and was used to monitor central venous pressure and obtain blood samples. We used a 60% oxygen-air mixture before and after OLV and an 80% oxygen-air mixture during OLV. Intraoperative ventilation was maintained using a pressure-controlled modality. The peak inspiratory pressure (PIP) was adjusted to provide a tidal volume of 8-10 mL/kg, and a positive end-expiratory pressure (PEEP) of 5 cm H2O was applied at all mechanical ventilation times for all children. Mechanical ventilation settings in the total lung ventilation (TLV) and OLV were determined according to the child's age and weight based on pediatric rates, rules, and tables for tidal volume (Vt), respiratory rate (RR), I: E ratio, and FiO_2_. The respiratory rate was titrated to achieve EtCO_2_ of 30-35 mmHg with TLV bilateral lung ventilation, and the settings were readjusted during OLV to maintain EtCO_2_ of 35-40 mmHg.

Time points of collecting arterial blood gas samples

The following assessment time points were followed:

T1: after 10 minutes of anesthetic induction and before OLV. This is the point at which the pulmonary shunt value, as affected by anesthesia, is calculated.

T2: 10 minutes after starting lung isolation and OLV. The anesthesia-affected pulmonary shunt value is obtained here, preceding the HPV effect that requires 15 minutes to develop.

T3: 60 minutes after OLV. The anatomical shunt fraction was calculated to assess the impact of HPV on the shunt. Intergroup comparisons were made.

T4: 20 minutes after the end of OLV, returned to full TLV bilateral lung ventilation.

**Figure 1:**
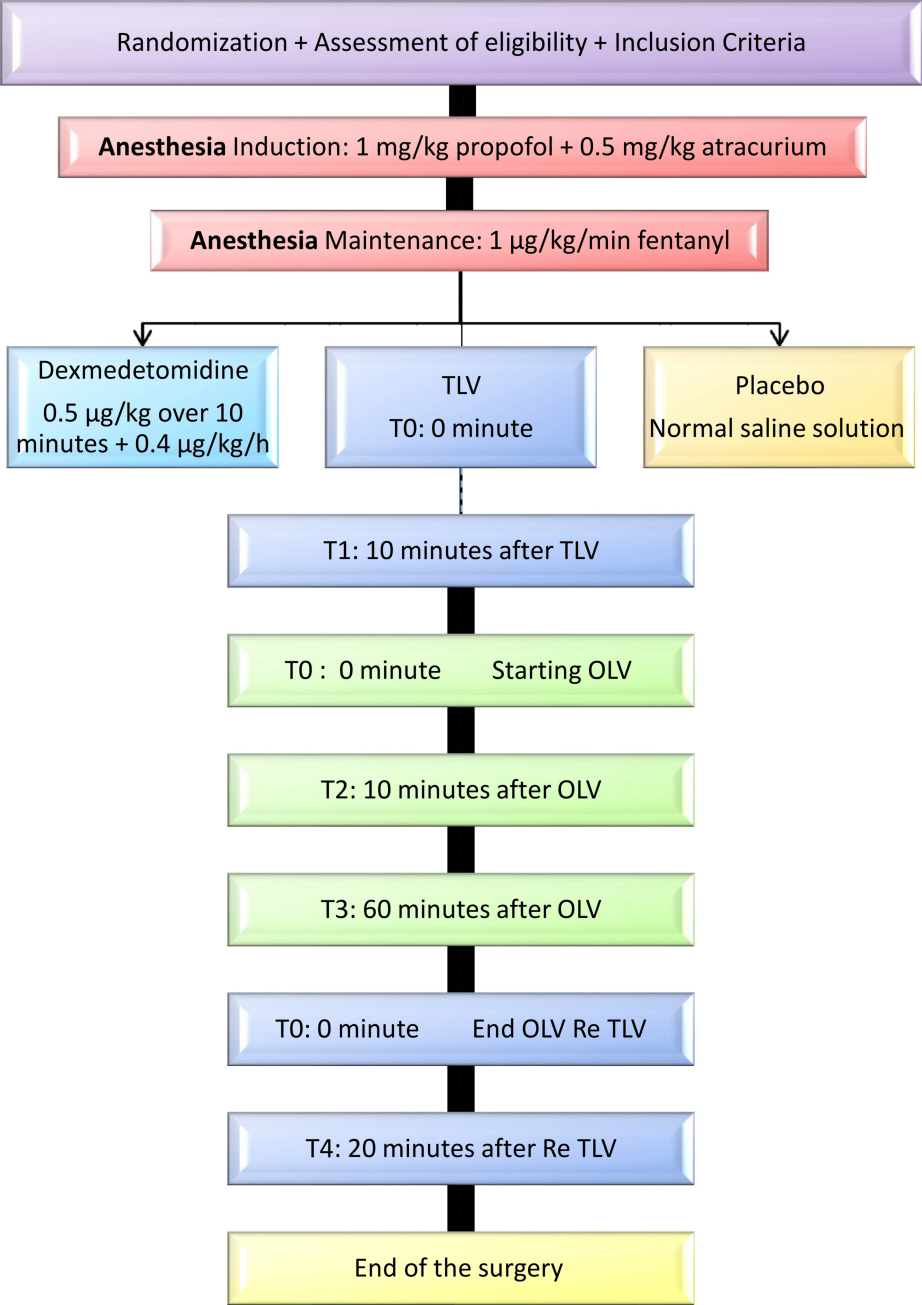
Stages of the experiment and the times for taking blood samples Times points: T1, T2, T3, T4 TLV: two-lung ventilation; OLV: one-lung ventilation; Re TLV: re-establishment of two-lung ventilation

Two arterial samples were collected each time: the first from the radial artery or aortic artery and the second from the pulmonary artery or right atrium through the central catheter. Figure 1 presents the stages of the trial and the times of blood sampling for arterial blood gas (ABG) analysis during surgery.

Outcome measures

The principal outcome metric employed in this study was the intraoperative variation observed in two key physiological parameters: arterial partial pressure of oxygen (PaO_2_) and the pulmonary shunt fraction (Qs/Qt).

The value of Qs/Qt was calculated according to the equations described by several previous studies [17]. The pulmonary shunt fraction was calculated as the difference between CaO_2_ and CcO_2_ divided by the difference between CvO_2_ and CcO_2_, where Qs is the shunted blood flow, Qt is the total cardiac output, CcO_2_ is pulmonary capillary oxygen content, CaO_2_ is arterial oxygen content, and CvO_2_ is mixed venous oxygen content.

The CcO_2_ represented the idealized oxygen content within the pulmonary capillaries; this value assumes complete oxygen equilibration between the alveoli and the capillary blood, signifying perfect gas exchange. CaO_2_ represented the oxygen content measured in the arterial blood, reflecting the oxygen delivered to the systemic circulation after leaving the left ventricle. CvO_2_ represented the oxygen content of the blood returning to the right atrium, a mixture of venous blood from the entire body, offering insight into the overall oxygen extraction at the tissue level [18].

Randomization and blinding

A departmental secretary not involved in this research project randomly allocated patients to the two groups. All injections were randomly prepared by anesthesia technicians not engaged in the study, and non-distinctive infusion pumps were prepared and then given to an anesthesiologist (with more than ten years of experience in pediatric anesthesia) without knowing the contents of the syringe. Allocation concealment was ensured through the implementation of a sealed envelope methodology. Both patients and attending anesthesiologists remained blinded to the treatment allocation (dexmedetomidine or placebo). Furthermore, the blinding protocol extended to graduate student researchers, the patient's families, and the patients themselves, ensuring that no individual involved in the study knew the specific group to which each child was allocated. The syringes were prepared according to the volume in milliliters, and the drug substance of each syringe was blinded to the medical team. Infusion syringes were prepared with a concentration of 0.25 μg/mL (dexmedetomidine or placebo) in 80 μg/20 mL, 200 μg/50 mL, or 400 μg/100 mL syringes.

Statistical analysis

Under the null hypothesis, it was hypothesized that no significant difference would be detected between the oxygen transport and pulmonary shunt groups concerning PaO_2_ and Qs/Qt. Statistical tests were performed to detect significant differences between the two groups using an independent-sample t-test. The data were analyzed using SPSS Version 27 (IBM Corp., Armonk, NY, USA). For this study, a significance level (alpha) of 0.05 was established, whereby a P-value below this threshold was interpreted as statistically significant.

## Results

Patient recruitment and follow-up

**Figure 2:**
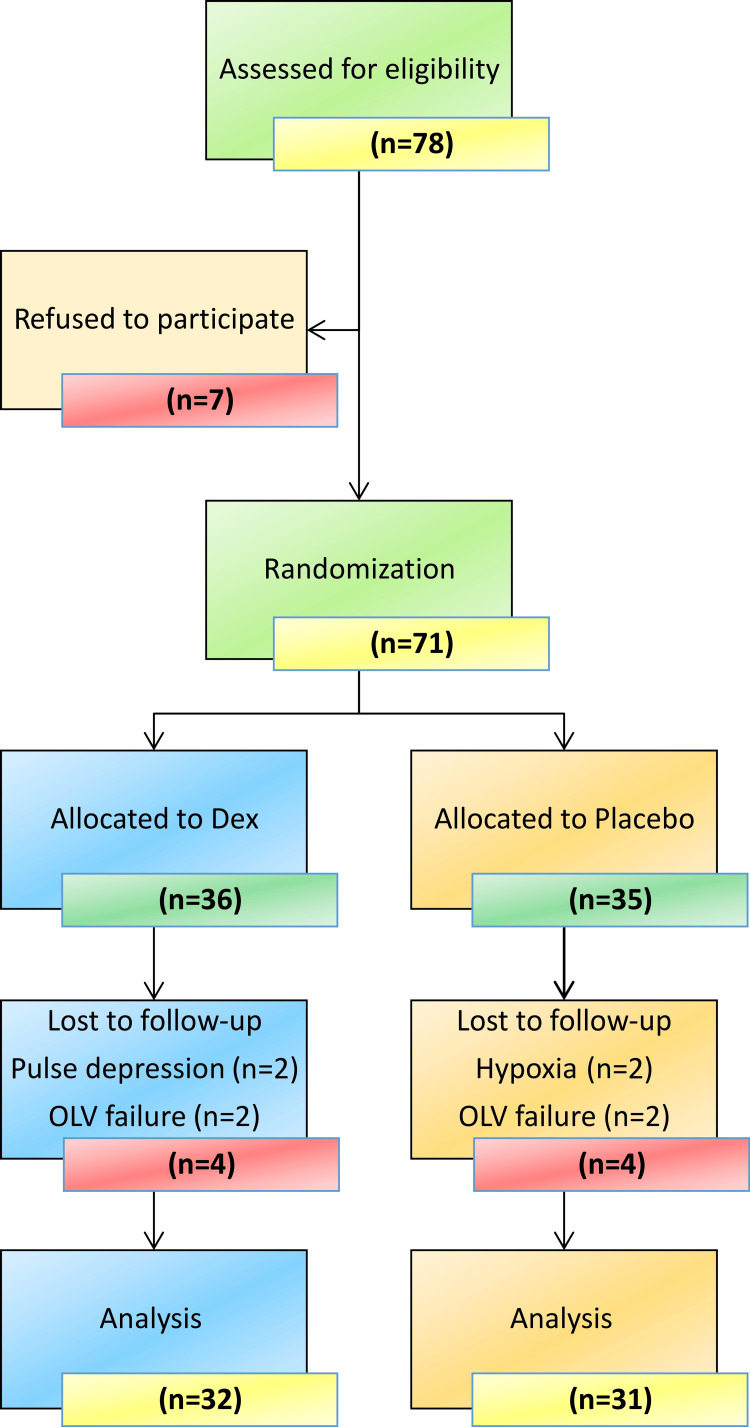
Flow diagram of patient recruitment, follow-up, and entry into data analysis TLV, two-lung ventilation; OLV, one-lung ventilation; Dex, dexmedetomidine

The study included 78 children who satisfied the requirements for enrollment were enrolled, of whom seven refused to participate, leaving 71 patients randomized to two groups. After obtaining written informed consent from parents or legal guardians on the day before surgery, dexmedetomidine (DEX group n = 36) or normal saline (placebo group n = 35) during anesthesia. Allocation was concealed from the researchers and participants. Four subjects in the DEX group withdrew from the experiment: two owing to failure of isolation and OLV administration and two due to low HR. Four placebo volunteers also discontinued the study: two due to failures in isolation and OLV administration and two due to decreased oxygen saturation. Finally, 32 participants in the DEX group and 31 in the placebo group completed the trial. A flow diagram of patient recruitment and follow-up is presented in Figure 2.

Demographic and baseline characteristics

The DEX and placebo groups exhibited no statistically significant differences in baseline characteristics (P > 0.05). Demographic attributes, including age, sex, weight, height, and body mass index (BMI), were comparable between groups. Surgical parameters, such as surgical duration and fluid administration, were similar. Furthermore, baseline physiological measures, including ABG values (pH, PaO_2_, PaCO_2_, hemoglobin), HR, MAP, and pulmonary function tests (FEV1/FVC), as well as the pulmonary shunt fraction (Qs/Qt), showed no significant intergroup differences. This meticulous matching of the groups effectively minimized the impact of potential confounders, strengthening the study's internal validity and allowing for a focused evaluation of the dexmedetomidine intervention (Table 1).

**Table 1:** Demographic and baseline data for all patients in the two groups with the p-value of significance testing *Independent two-sample t-test was used for continuous variables (e.g., age, weight, BMI, height, operation time, crystalloid, colloid, urine output, pH, PaO_2_, PaCO_2_, hemoglobin, FEV1/FVC, HR, MAP) and chi-square test was used for categorical variables (e.g., gender). A low P-value (generally below 0.05) indicates a statistically significant difference between the two groups. DEX, dexmedetomidine; BL, baseline; PaO_2_, partial pressure of oxygen in arterial blood; PaCO_2_, partial pressure of carbon dioxide; Hg, hemoglobin; HR, heart rate; MAP, mean arterial pressure; FEV1, forced expiratory volume in 1 second; FVC, forced vital capacity

Variable	DEX (n=32), mean (SD)	Placebo (n=31), mean (SD)	P-value*
Age	4.95 (0.57)	4.90 (0.80)	0.81
Gender = male (%)	15 (46.9%)	12 (38.7%)	0.689
Weight (kg)	16.73 (1.41)	16.26 (1.80)	0.247
Height (cm)	110.03 (8.76)	106.74 (10.21)	0.175
BMI	16.73 (1.40)	16.26 (1.79)	0.958
Operation time (min)	113.31 (25.29)	115.39 (26.03)	0.749
Crystalloid (mL/kg)	10.17 (3.18)	9.26 (3.11)	0.253
Colloid (mL/kg)	7.13 (2.43)	7.52 (2.18)	0.501
Urine output (mL/kg)	2.88 (0.82)	2.76 (0.74)	0.56
Hb	11.88 (1.10)	11.86 (1.14)	0.935
FEV1/FVC	75.28 (2.18)	75.44 (1.83)	0.747
pH	7.40 (0.02)	7.40 (0.02)	0.564
PaO_2_ (mmHg)	74.50 (4.96)	75.26 (4.86)	0.542
PaCO_2_ (mmHg)	36.97 (1.18)	37.26 (1.67)	0.429
HR (beats/min) (BL)	133.33 (3.44)	134.58 (3.24)	0.143
MAP (mmHg) (BL)	72.23 (6.31)	72.42 (8.33)	0.918

Intraoperative changes in various parameters for each group are presented in Table 2. We want to clarify that all children enrolled in this trial were ASA class III patients with pre-existing medical records, including results from various medical tests such as pulmonary function tests and ABG analysis. These records also included prior Qs/Qt measurements performed by their pediatricians as part of their routine medical care and anesthetic risk assessment. Therefore, the baseline Qs/Qt values reported in this study were obtained retrospectively from these medical records.

**Table 2:** Descriptive statistics of the collected variables in the two groups across the different assessment times Mean, the average value for each variable in each group at each time point; SD, standard deviation; P-value, the result of a statistical test comparing the dexmedetomidine and placebo groups for each variable at each time point. *Independent two-sample t-test was used for continuous variables (PaO_2_, PaCO_2_, Qs/Qt, HR, MAP). A low P-value (below 0.05) indicates a statistically significant difference between the two groups. Baseline: The first measurements made before administering medication or introducing any intervention. The different time points during surgery: T1 TLV 10 (two-lung ventilation after 10 minutes), T2 OLV 10 (OLV after 10 minutes), T3 OLV 60 (OLV after 60 minutes), T4 Re TLV (re-establishment of TLV) Dex, dexmedetomidine; HR, heart rate; MAP, mean arterial pressure; OLV, one-lung ventilation; PIP, peak inspiratory pressure; PaO_2_, partial pressure of oxygen in arterial blood; PaCO_2_, partial pressure of carbon dioxide; Qs/Qt, pulmonary shunt fraction; TLV, two-lung ventilation

Groups		Baseline				T1 TLV 10				T2 OLV 10				T3 OLV 60				T4 Re TLV			
		Mean	SD	SEM	P-value*	Mean	SD	SEM	P-value*	Mean	SD	SEM	P-value*	Mean	SD	SEM	P-value*	Mean	SD	SEM	P-value*
PaO_2_ (mmHg)	Dex	74.5	5	0.88	0.54	313.4	34.96	6.18	0. 975	207.8	46.7	8.25	0.001	234.8	40.4	7.14	<0.001	305.7	37.1	6.56	0. 792
	Placebo	75.26	4.9	0.87		313.7	33.2	5.96		168.3	41.7	7.49		192.2	49.6	8.9		303.2	36.6	6.56	
Qs/Qt	Dex	12.41	1.4	0.25	0.74	15.2	2.263	0.4	0. 895	23.96	2.77	0.49	<0.001	21.64	3.36	0.59	<0.001	19.59	0.59	0.1	<0.001
	Placebo	12.29	1.3	0.23		15.27	1.762	0.32		29.23	3.33	0.6		26.94	1.1	0.2		21.86	1.14	0.21	
PIP (cmH_2_O)	Dex					14.56	1.045	0.19	0. 413	19.81	2.92	0.52	<0.001	17.09	3.22	0.57	<0.001	16.88	4.7	0.83	0. 790
	Placebo					14.77	0.99	0.18		23.84	4.46	0.8		24.9	4.17	0.75		17.19	4.76	0.85	
HR (beats/min)	Dex	133.3	3.4	0.61	0.14	114.3	6.542	1.16	0. 668	119.4	17.1	3.02	0.363	118.3	20.7	3.67	0. 239	110.8	11.2	1.98	0. 657
	Placebo	134.6	3.2	0.58		115	7.003	1.26		123.7	20.8	3.61		124	17.2	3.09		112.3	14.3	2.57	
MAP (mmHg)	Dex	72.23	6.3	1.12	0.92	66.32	8.313	1.47	0. 299	68.5	12.1	2.14	0.692	69.94	10.4	1.84	0. 879	70.72	9.86	1.74	0. 795
	Placebo	72.42	8.3	1.5		68.65	9.344	1.68		69.79	13.6	2.45		70.34	10.3	1.85		70.08	9.64	1.73	

Partial pressure of arterial oxygen (PaO_2_)

**Figure 3:**
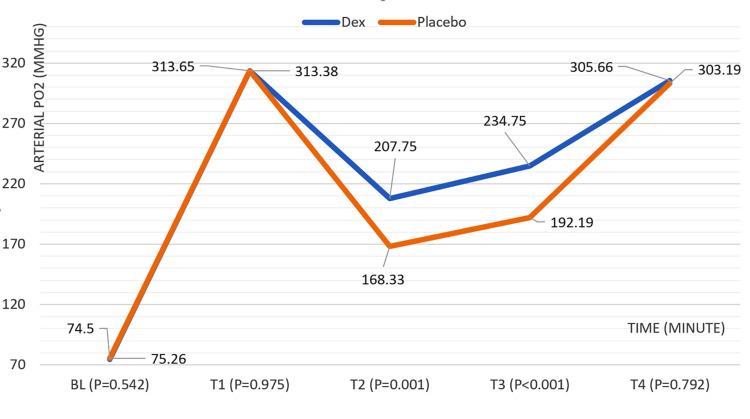
Arterial PaO2 (mmHg) changes in children during surgery according to time (minute) and the groups. PaO_2_, partial pressure of oxygen; Dex, dexmedetomidine

Baseline and initial PaO_2_ values showed no significant differences between the DEX and placebo groups (P = 0.542 and P = 0.975, respectively). While PaO_2_ decreased in both groups after OLV, the DEX group maintained significantly higher PaO_2_ levels at later stages (T2: P < 0.001; T3: P < 0.001). This difference disappeared upon resuming double-lung ventilation (T4: P = 0.792). Figure 3 illustrates the temporal changes in PaO_2_ throughout the surgical procedure for both groups.

Ratio of pulmonary shunt fraction (Qs/Qt)

**Figure 4:**
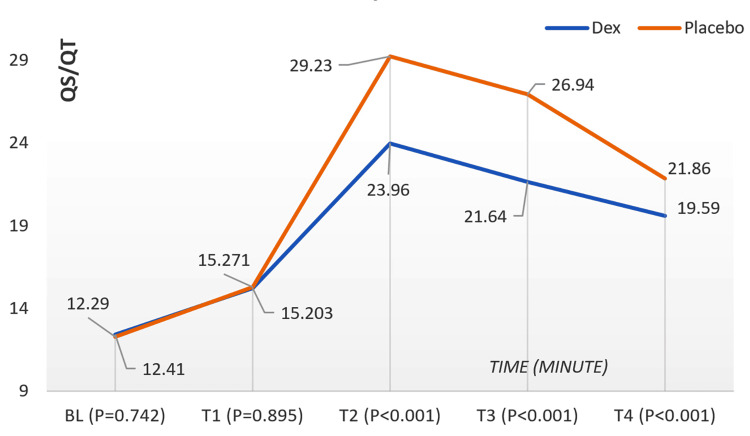
Qs/Qt changes in children during surgery according to time (minute) and the groups. Qs/Qt, ratio of pulmonary shunt fraction; Dex, dexmedetomidine

No significant group difference in (Qs/Qt) was observed at baseline (P = 0.742) or T1 (P = 0.895). While Qs/Qt increased post-OLV in both groups, it remained significantly lower in the DEX group than in placebo at all subsequent time points, persisting even after OLV discontinuation (T2-T4, P < 0.001). Figure 4 depicts the temporal changes in Qs/Qt during surgery, stratified by group. In pediatric thoracic surgery, dexmedetomidine appeared to improve oxygenation during OLV by reducing the pulmonary shunt fraction (Qs/Qt), compared to placebo.

Peak inspiratory pressure

**Figure 5:**
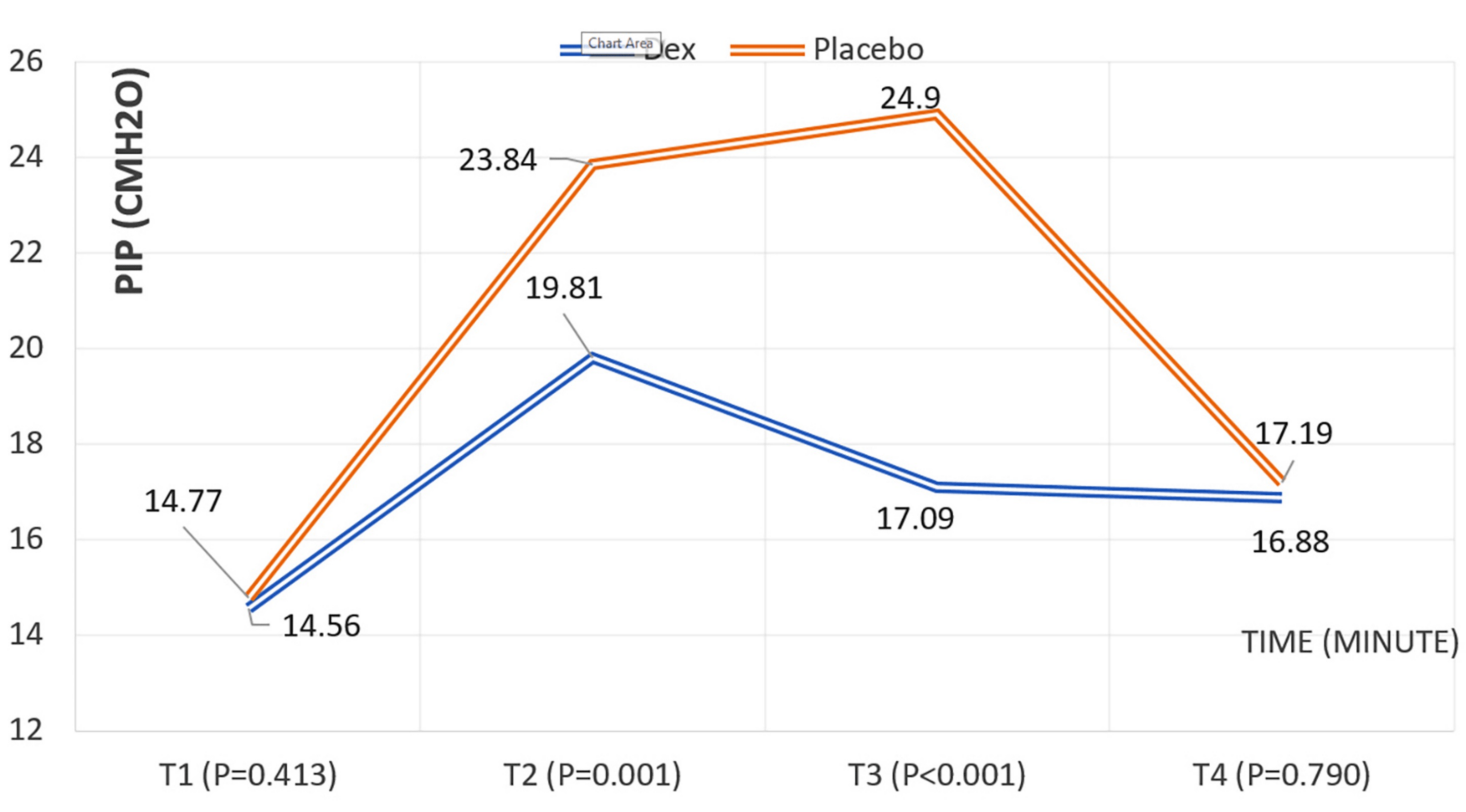
PIP (cmH2O) changes in children during surgery according to time (minute) and the groups PIP, peak inspiratory pressure; Dex, dexmedetomidine

No difference in PIP was observed between groups during two-lung ventilation (T1, P = 0.413). With lung isolation and OLV, PIP increased in both groups; however, the DEX group had significantly lower PIP than placebo (T2: P < 0.001; T3: P < 0.001). Following OLV discontinuation, PIP values returned to similar levels between groups (T4: P = 0.790). Figure 5 illustrates these changes in PIP throughout surgery.

Mean arterial pressure

No difference in MAP was observed between groups at baseline (P = 0.918). While MAP decreased slightly (<10%) from baseline in both groups after anesthesia induction, this difference was not statistically significant (T1, P = 0.299). Throughout the remaining surgery, including OLV and TLV, MAP remained similar between groups (P > 0.05).

Heart rate

No baseline difference in HR was observed between groups (P = 0.143). This similarity persisted throughout the entire surgery, with no statistically significant differences observed between groups at any time point (P > 0.05).

Complications

No serious complications were reported during the study. A slight decrease in blood pressure (<10%) was observed in some children in both groups following anesthesia induction; however, this was not statistically significant and did not require intervention. Two patients in the DEX group were excluded due to persistent bradycardia despite atropine administration, which required stopping the drug (dexmedetomidine) infusion. Two patients in the placebo group were excluded from the study due to persistent oxygen desaturation (<90%) despite standard interventions to improve oxygenation, necessitating the abandonment of OLV.

## Discussion

This study contributes to the developing body of literature exploring the effects of dexmedetomidine on lung function during OLV in pediatric patients. Building upon the foundation established by previous investigations primarily conducted on adult populations [19], we sought to evaluate the efficacy of dexmedetomidine in improving oxygenation and reducing intrapulmonary shunting in children aged four to six years undergoing thoracic surgical procedures requiring OLV.

While no prior pediatric studies existed within this context, our trial adopted a dexmedetomidine dosing regimen informed by existing literature on pediatric dexmedetomidine pharmacokinetics and pharmacodynamics [12,20,21]. The chosen loading dose of 0.5 μg/kg over 10 minutes, followed by a maintenance infusion of 0.4 μg/kg/hour, aimed to balance potential therapeutic benefits while minimizing the risk of adverse hemodynamic effects in this vulnerable population.

Our findings unequivocally demonstrate that dexmedetomidine administration significantly improves the intrapulmonary shunt ratio (Qs/Qt) compared to the control group. This improvement in Qs/Qt, a sensitive indicator of intrapulmonary shunting [19], translates into a statistically significant enhancement in blood oxygen level (PaO_2_) during OLV. These results resonate with Wang et al.'s study [19], which, albeit using a different dosing regimen in adults, reported a significant reduction in Qs/Qt with dexmedetomidine. These findings underscore dexmedetomidine's potential to optimize oxygenation during OLV, a critical aspect of managing patients undergoing thoracic surgical interventions.

The mechanisms underlying dexmedetomidine's beneficial effects on lung function during OLV are likely multifactorial. Firstly, dexmedetomidine's capacity to enhance HPV has been well-documented [10,14]. This physiological response, crucial for maintaining ventilation-perfusion matching, involves redirecting blood flow away from poorly ventilated lung regions towards better-ventilated areas, thereby improving oxygenation [10]. Dexmedetomidine's ability to regulate the expression of vasoactive substances likely contributes to this augmented HPV response [14]. Furthermore, dexmedetomidine diminishes the shunt fraction by effectively reducing intrapulmonary shunt, further contributing to enhanced gas exchange efficiency.

Secondly, while not a primary focus of the current investigation, it is plausible that dexmedetomidine exerts positive effects on lung mechanics [14,22]. Evidence suggests that dexmedetomidine can improve lung compliance and reduce airway resistance, facilitating lung expansion and promoting optimal ventilation [14,22]. However, it is crucial to acknowledge that significant bronchodilatory effects and alterations in lung compliance are predominantly observed at higher plasma concentrations of dexmedetomidine [20,21]. Given the relatively low dose used in our study and the observation that children in this age group exhibit lower susceptibility to dexmedetomidine's pulmonary effects compared to younger patients [20], we posit that the likelihood of impacts on bronchodilation and lung compliance in our groups is minimal. Nevertheless, the observed reduction in PIP in the DEX group warrants further investigation to delineate the potential influence of dexmedetomidine on lung mechanics in this population.

Thirdly, dexmedetomidine's ability to attenuate the inflammatory response associated with OLV constitutes another plausible mechanism contributing to its beneficial effects [23,24]. Multiple studies have demonstrated dexmedetomidine's efficacy in reducing serum concentrations of pro-inflammatory cytokines such as interleukin-6 (IL-6) and tumor necrosis factor-alpha (TNF-α) [23-25]. By mitigating the inflammatory cascade triggered by OLV, dexmedetomidine may play a crucial role in preserving lung function and preventing the development of postoperative pulmonary complications.

Beyond the aforementioned mechanisms, other potential contributors to dexmedetomidine's pulmonary benefits have been proposed, including the stimulation of nitric oxide (NO) release leading to pulmonary vasodilation [22] and the counteraction of the inhibitory effects of certain anesthetic agents on HPV [14]. The positive impact of dexmedetomidine on pulmonary outcomes in thoracic surgery involving OLV has been consistently demonstrated in numerous studies and meta-analyses [14,22]. Our findings, characterized by improved PaO_2_ and reduced Qs/Qt with dexmedetomidine, align seamlessly with this growing body of evidence.

Furthermore, our results corroborate the findings of Asri et al. Xu et al., Liu et al., and Yang et al. [14,26-28] reported statistically significant improvements in PaO_2_ during OLV with dexmedetomidine administration. Similarly, Jiang et al.'s study [29] demonstrated that dexmedetomidine can mitigate the inflammatory response, oxidative stress, and oxygen requirements while concurrently enhancing Qs/Qt and PaO_2_ during OLV. These converging lines of evidence strongly suggest that dexmedetomidine may constitute a valuable therapeutic intervention to combat hypoxia during lung surgery, potentially leading to improved postoperative outcomes and reduced hospital stays [22].

While our study did not reveal statistically significant differences in hemodynamic variables between the DEX and control groups, it is important to consider potential explanations for this observation. The relatively low dose of dexmedetomidine employed in our investigation, consistent with recommendations for pediatric patients [12,21,30,31], may have contributed to the absence of significant hemodynamic effects. Indeed, the dose-dependent nature of dexmedetomidine's hemodynamic influence has been previously documented [12,21,30,31].

Furthermore, the unique physiological characteristics of our pediatric groups, including age-related variations in drug metabolism and sensitivity, may have influenced their response to dexmedetomidine [20]. Further pharmacokinetic studies specifically tailored to pediatric populations are warranted to fully elucidate these aspects. Additionally, the co-administration of propofol, a widely used anesthetic agent with known hemodynamic effects [32], may have interacted with dexmedetomidine, potentially masking its subtle hemodynamic influence. This potential interaction warrants further investigation in a pediatric context.

Despite the lack of statistically significant hemodynamic effects in our study, the clear benefits observed for ventilation and oxygenation during lung isolation, evidenced by improved PaO_2_ and reduced Qs/Qt, underscore the positive impact of dexmedetomidine on pulmonary function that extends beyond hemodynamic parameters [14].

Limitations of the current study

This single-center study, conducted at Children's University Hospital, was designed to leverage the institution's expertise in pediatric thoracic surgery and OLV anesthesia. However, the relatively small sample size necessitates further investigation with larger and more diverse patient populations to validate our findings. Additionally, our exploration of a single dexmedetomidine dosage warrants further research to investigate alternative dosing regimens and compare their efficacy in pediatric patients. The inherent complexities of obtaining precise physiological measurements in children, particularly during OLV, pose a recognized challenge in this field. Moreover, the high cost and limited availability of dexmedetomidine present practical obstacles to conducting larger-scale studies. Securing additional funding support from grant agencies is crucial to facilitate more comprehensive research in this area.

## Conclusions

In high-risk pediatric thoracic surgery, dexmedetomidine infusion during OLV demonstrated several benefits, notably reduced shunt and pulmonary shunt fraction (Qs/Qt), leading to improved gas exchange and significantly enhanced PaO_2_ levels, reflecting increased blood oxygenation. It also decreased PIP, suggesting improved lung mechanics. Furthermore, dexmedetomidine maintained hemodynamic stability, including stabilization of MAP and HR. In conclusion, this study suggests that dexmedetomidine may be a valuable adjunct for managing OLV in pediatric thoracic surgery.
